# Supplementary material for: Deciphering neuronal deficit and protein profile changes in human brain organoids from patients with creatine transporter deficiency
Source: eLife. 2023 Oct 13;12:RP88459. doi: 10.7554/eLife.88459 (PMC10575631; doi:10.7554/eLife.88459)
Supplement: Figure 6—source data 5. [file elife-88459-fig6-data5.zip › Figure 6 - Source data 5/Figure 6 - Source data.pptx]

## Slide 1
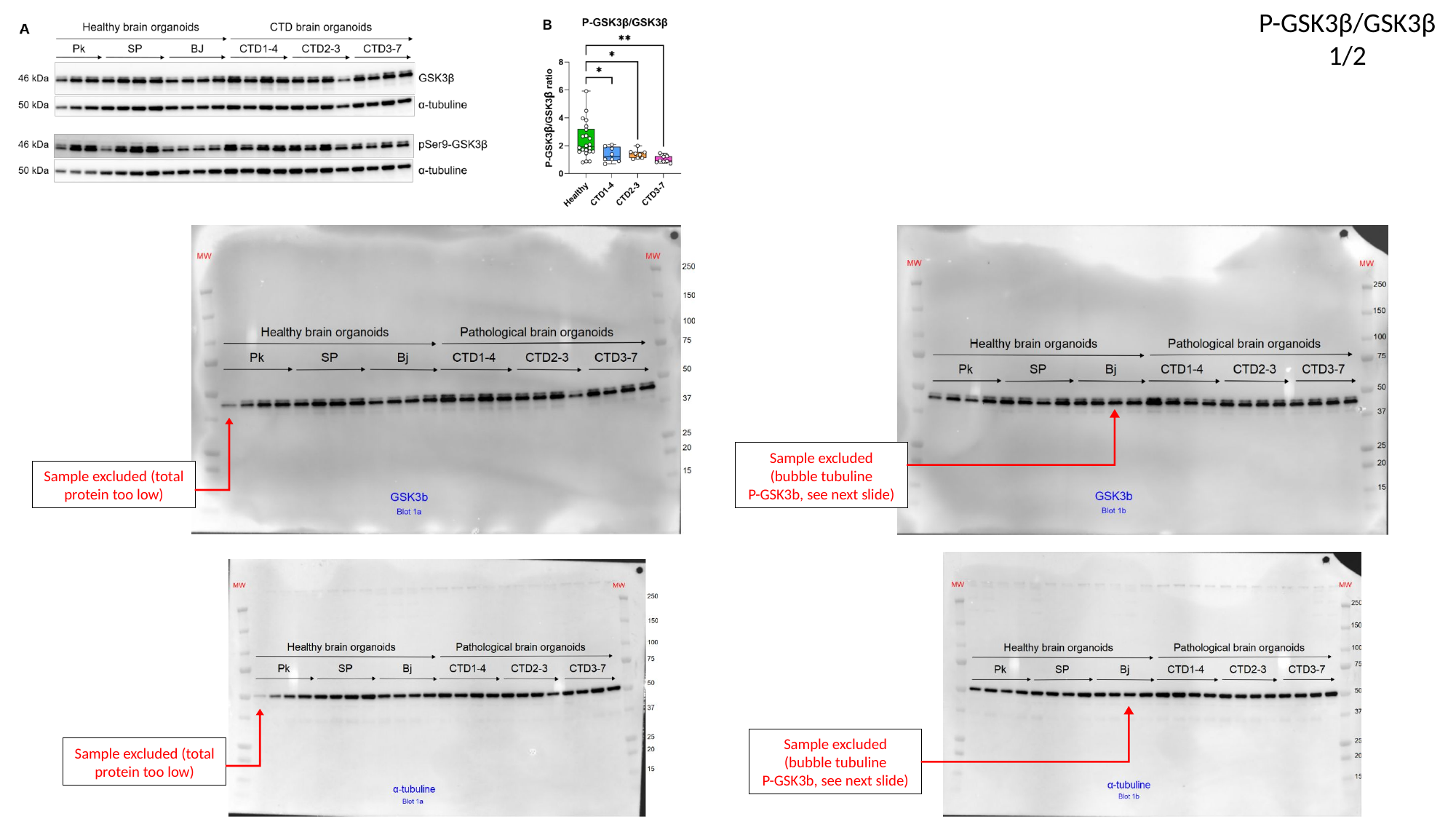

P-GSK3β/GSK3β
1/2
Sample excluded (total protein too low)
Sample excluded
(bubble tubuline
P-GSK3b, see next slide)
Sample excluded
(bubble tubuline
P-GSK3b, see next slide)
Sample excluded (total protein too low)

## Slide 2
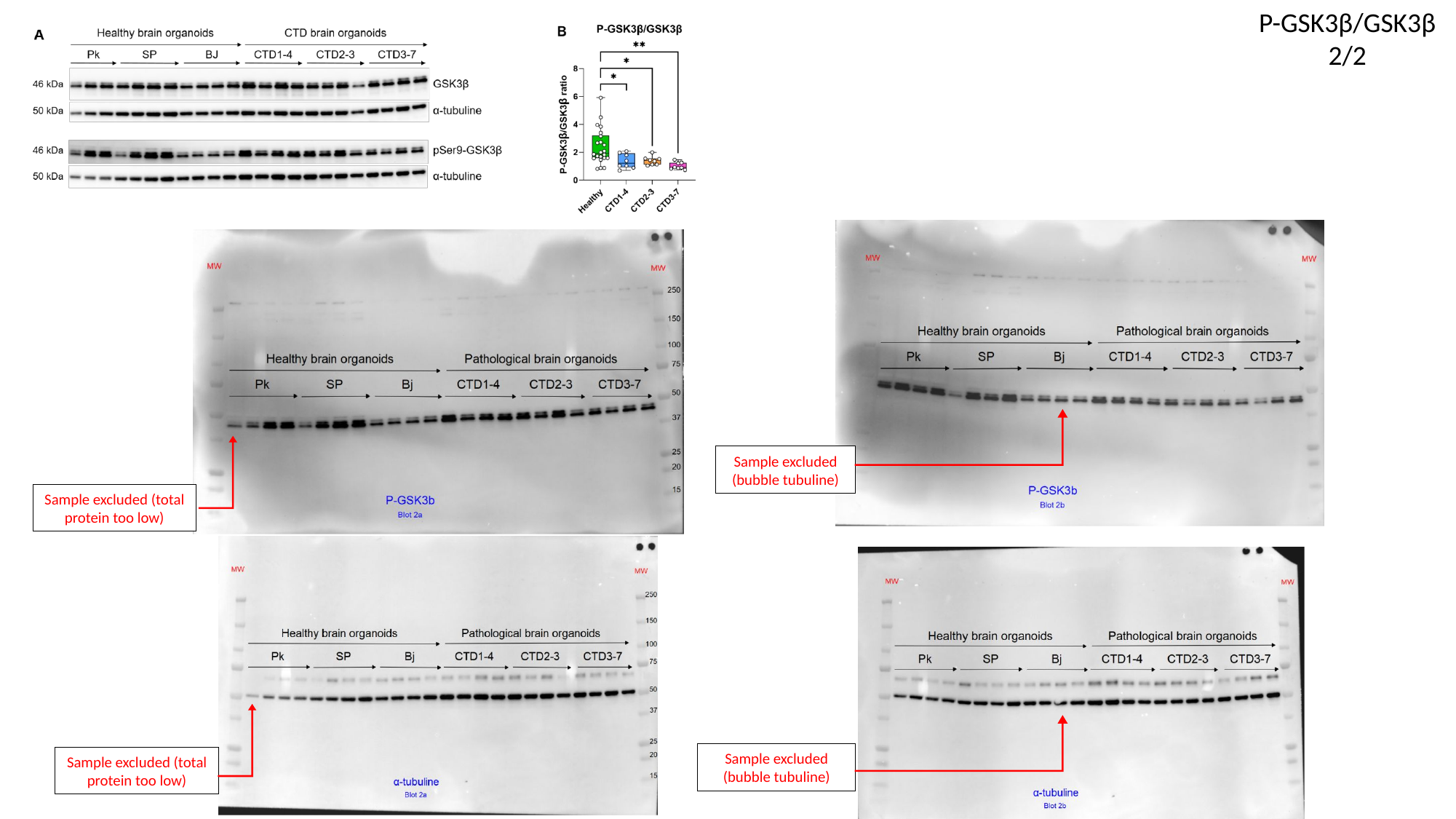

P-GSK3β/GSK3β
2/2
Sample excluded (bubble tubuline)
Sample excluded (total protein too low)
Sample excluded (total protein too low)
Sample excluded (bubble tubuline)

## Slide 3
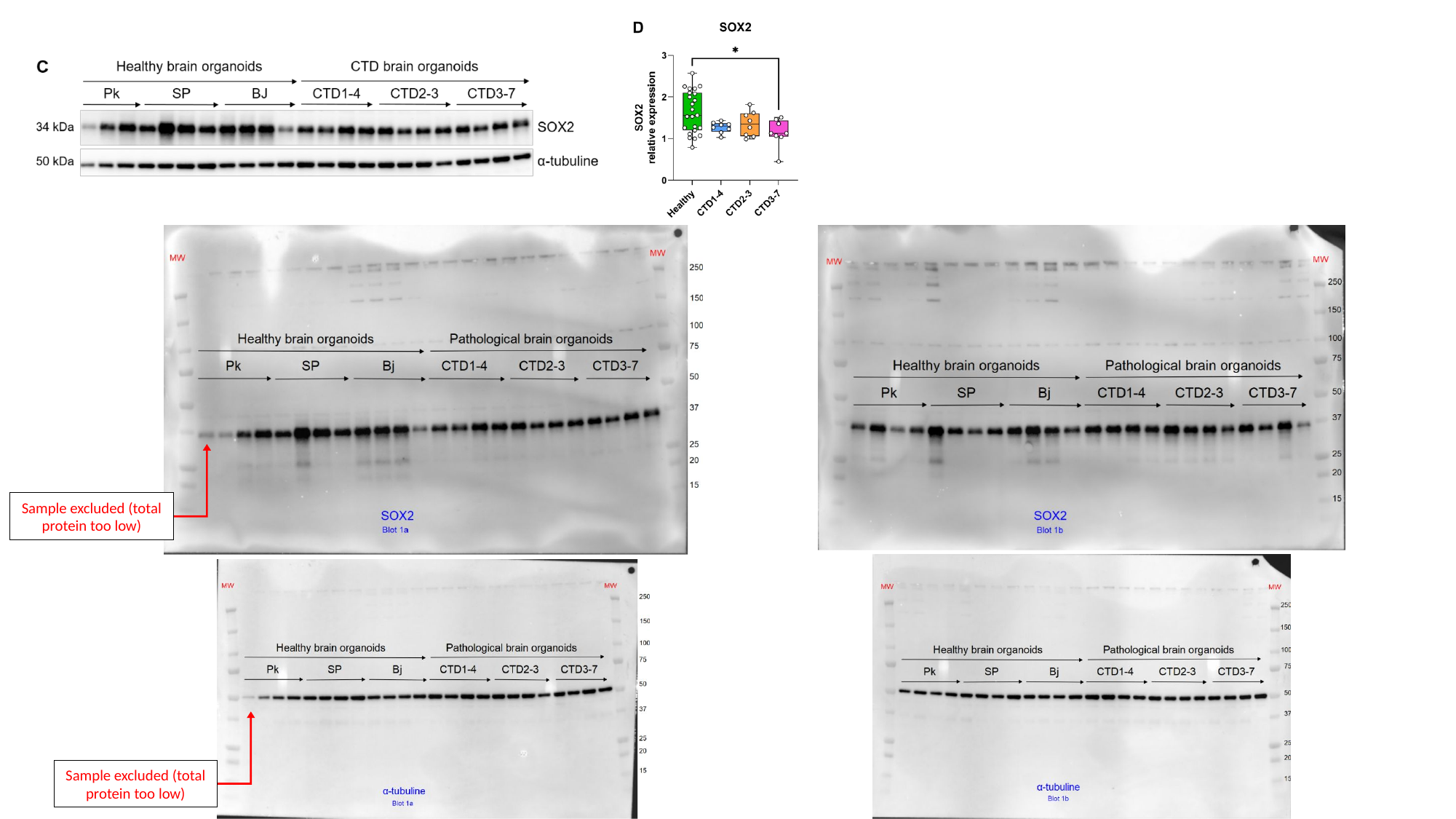

Sample excluded (total protein too low)
Sample excluded (total protein too low)

## Slide 4
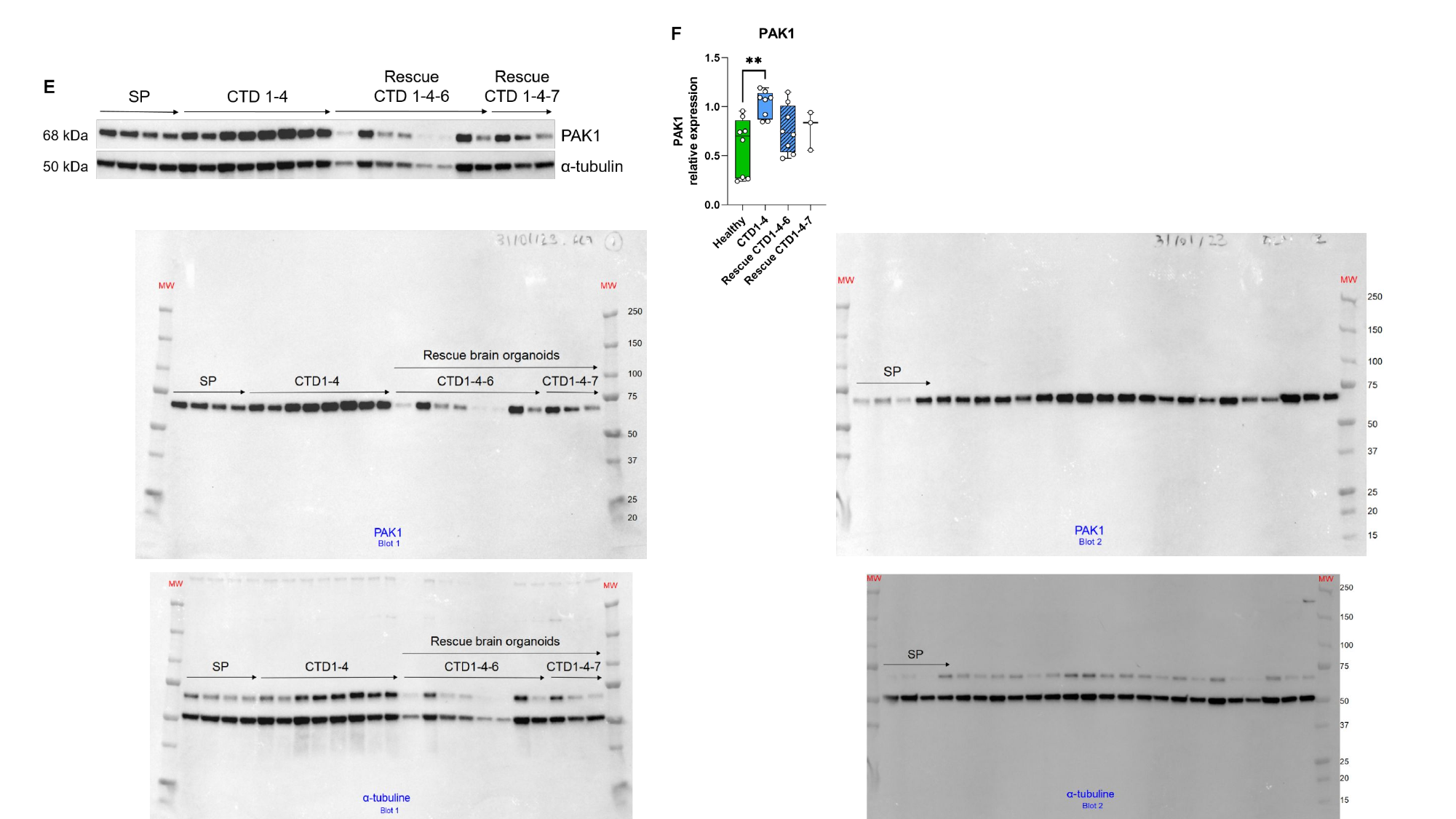

## Slide 5
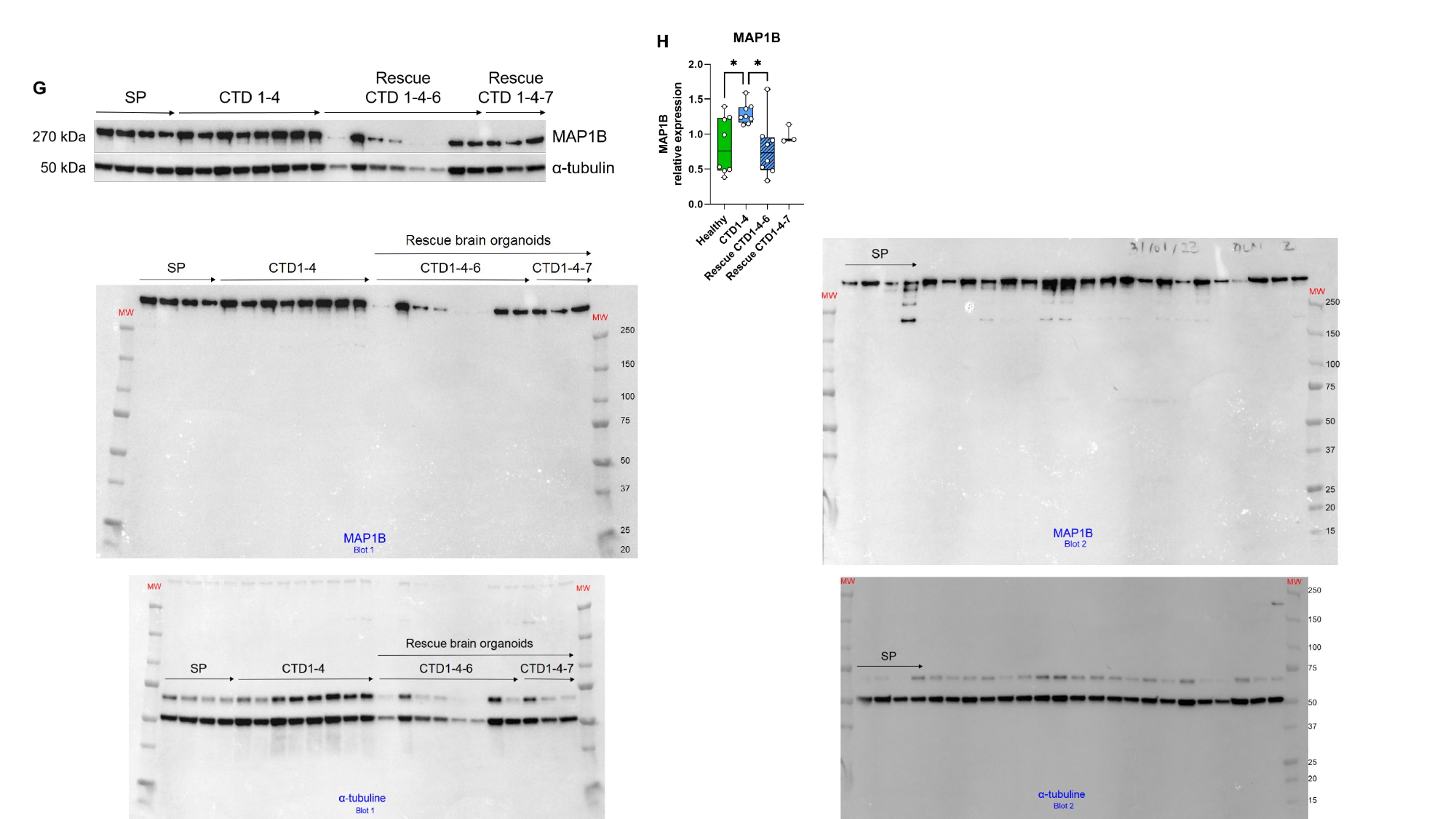

## Slide 6
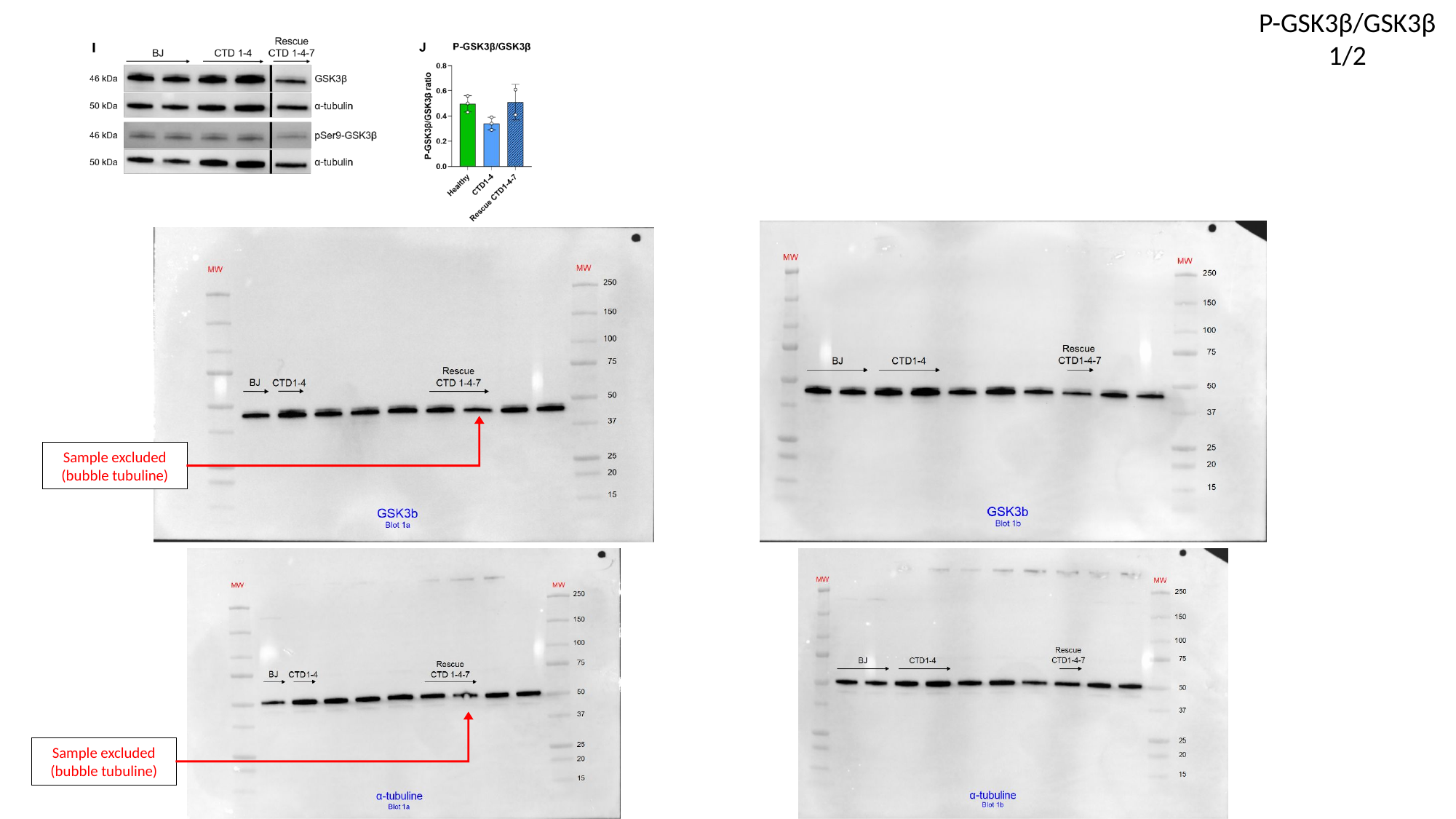

P-GSK3β/GSK3β
1/2
Sample excluded (bubble tubuline)
Sample excluded (bubble tubuline)

## Slide 7
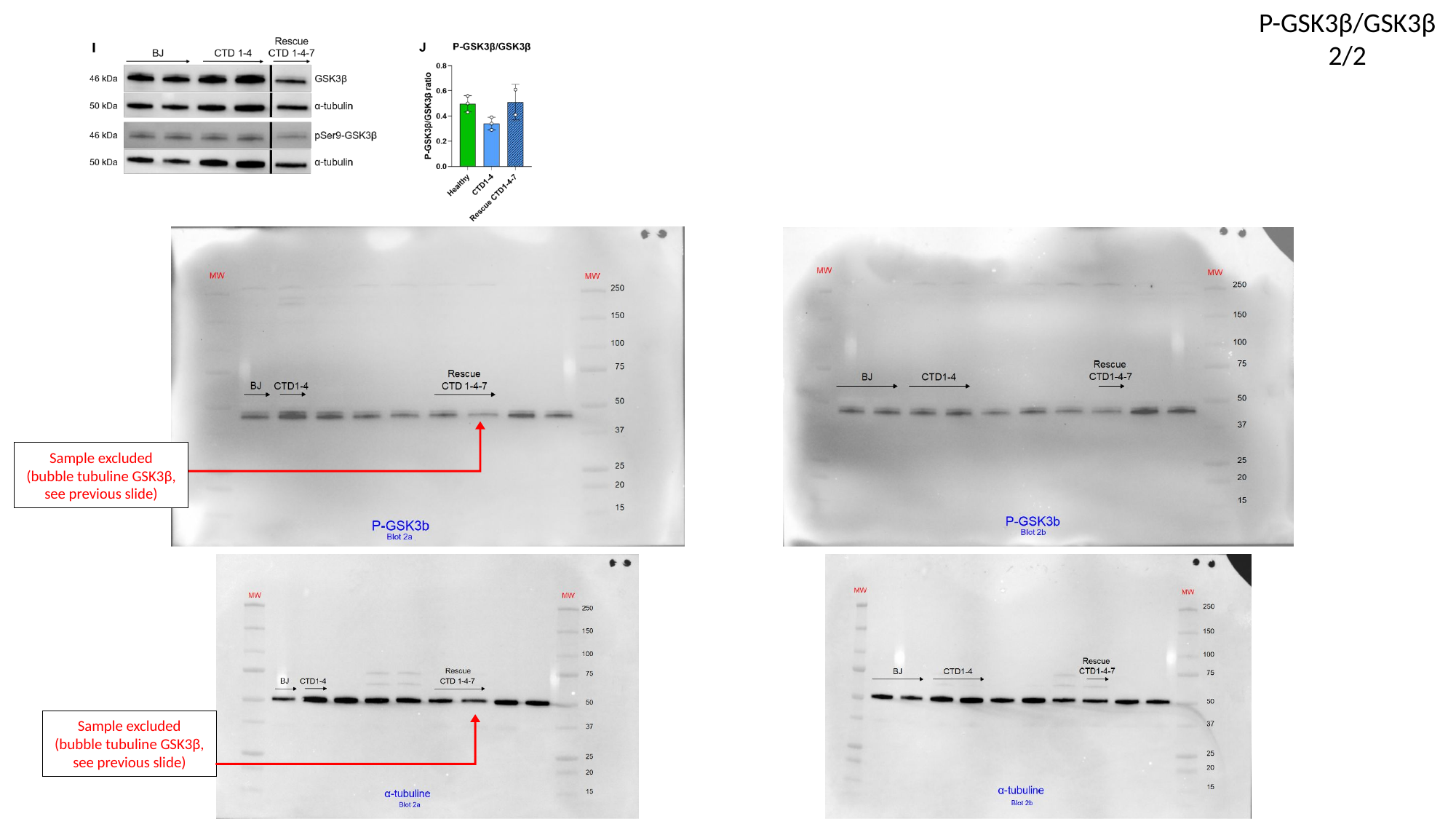

P-GSK3β/GSK3β
2/2
Sample excluded (bubble tubuline GSK3β, see previous slide)
Sample excluded (bubble tubuline GSK3β, see previous slide)

## Slide 8
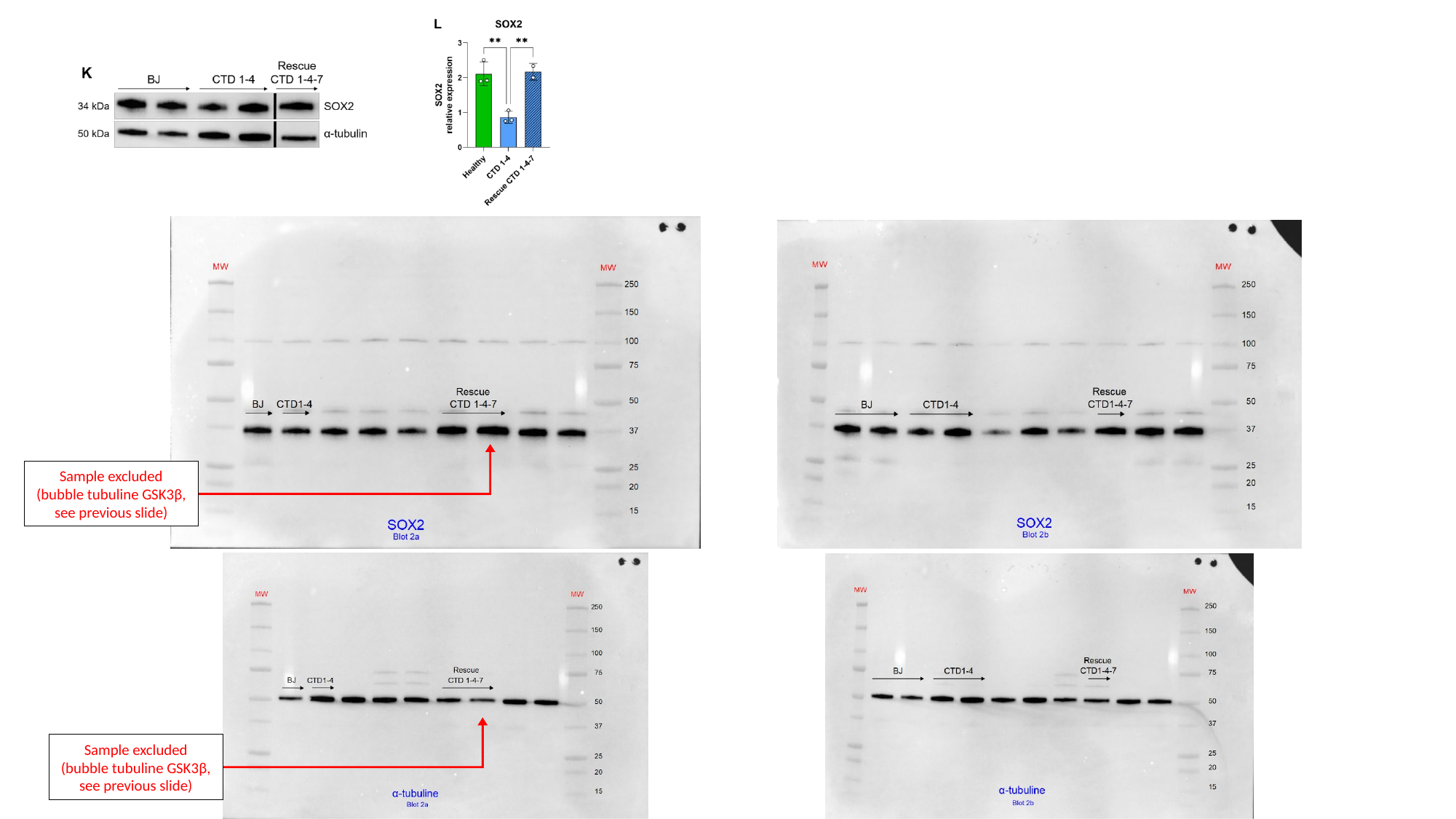

Sample excluded (bubble tubuline GSK3β, see previous slide)
Sample excluded (bubble tubuline GSK3β, see previous slide)
